# ‘Scrub up’: assessing how a gynaecology podcast can enhance the learning of medical students, a mixed-methods study

**DOI:** 10.1186/s12909-025-08293-2

**Published:** 2025-11-29

**Authors:** Lucy Richards, Tegan Grace, Graeme Horton, Bunmi Malau-Aduli, Craig Pennell

**Affiliations:** 1 Department of Maternity and Gynaecology, John Hunter Hospital, Newcastle, Australia; 2 School of Medicine and Public Health, University of Newcastle, Newcastle, Australia; 3 School of Rural Medicine, University of New England, Newcastle, Armidale Australia

**Keywords:** Podcast, Medical education, Gynaecology

## Abstract

**Background:**

Podcasts are widely used by medical students to keep up to date with knowledge, revise and prepare for examinations and medical placements. They are valued for their ease of access, asynchronous accessibility and relatable tone. In obstetrics and gynaecology, tools such as podcasts that enable flexible learning around demanding and changeable workloads are highly valuable, however research to understand how student learners utilise podcasts and their efficacy in this context is limited.

**Methods:**

This was a sequential exploratory mixed methods study with two phases. Phase one was a descriptive qualitative study using focus group discussions and semi-structured questions to explore student attitudes and preferences in relation to podcasts. Phase two of the study utilised data from phase one to develop an educational podcast with a gynaecological focus. Subsequently, students listened to the podcast and then completed an evaluation questionnaire that assessed their perceptions of the podcast design, content and effectiveness as a learning tool. The data were triangulated to draw conclusions about the overall utility of podcast as a learning tool for medical students in gynaecology.

**Results:**

Focus group data from 14 participants revealed five main themes relating to podcast usage and learning preferences. These were (1) ‘personal and relatable’ (2) ‘reflects real world clinical practice’ (3) ‘use alongside conventional study’ (4) ‘structured and focused content’ and (5) ‘targets what I need to know’. These themes informed the creation of a gynaecology podcast targeting student learning preferences. The podcast was made available to 201 students and utilised by 68. 92.7% of 55 respondents found the podcast effective or very effective at improving their knowledge in gynaecology topic areas. The majority (76.3%) of students felt podcasts should be included within the university medical program in the future.

**Conclusions:**

Our respondents regarded podcasts as an important learning platform that is highly accessible and engaging. Utilisation of key components of podcasts may assist content producers to create high yielding learning opportunities for students. Podcasts offer an alternative to conventional educational delivery that can offer students flexibility and engaging content that may be tailored to suit the specific learning needs of medical students.

## Background

Podcasts are media files (traditionally audio, but more recently also video files known as ‘vodcasts’) with recorded content that are often produced in a series, available to stream or download to a user’s smartphone or computer. In recent times, there has been an exponential rise in free open access education (FOAM) materials for health professionals which include podcasts along with blogs and websites [1]. Medically relevant podcasts are utilised by up to 35–88% of all medical residents [2] and increasingly this usage is expanding into medical educational institutions with one medical school in the United States (US) integrating podcasts into formal curriculum [3].

Driven by pressures of a global pandemic, ever expanding knowledge base and changing landscape of technology in healthcare and education, the delivery of medical education must fundamentally shift to stay abreast of this change. Podcasts have been reported to have many benefits when compared with conventional education modalities such as reading textbooks or journals [4]. These benefits include the ease and flexibility in access, ability for learners to engage with content at their own pace [2] and the casual tone that listeners find not only engaging but enjoyable [5]. Uniquely, podcasts can connect listeners to experts in the field across the globe in an environment that is relatable and not intimidating [6].

Podcasts have many benefits for learning in medical specialties such as obstetrics and gynaecology where flexibility and ease of access is required around rostered after-hours, shift work and fluctuating workflow. Despite this, compared with other specialties, obstetrics and gynaecology focused podcasts are under-represented [7]. One review in 2020 reported that are only 10 active podcasts in obstetrics and gynaecology with one podcast providing over 32% of the total number of episodes [8] A more recent 2024 systematic review reports podcasts in obstetrics and gynaecology make up only 7% of all medical education podcasts [9].

Research into the effectiveness of podcasts as a learning tool in obstetrics and gynaecology is limited. There is some evidence that podcasts provide equivalent knowledge retention for medical students to conventional lecture formats [7] and this has been confirmed in a gynaecological context in one small study assessing a podcast focused on vulval disorders [10]. The current lack of governance and oversight for podcast content is also of concern. One study revealed that the majority of podcasts were produced by unaffiliated individuals or companies rather than traditional medical educational institutions such as universities or professional societies [11].

While podcasts have the potential to be used effectively as a mode of education within our medical institutions, more research is needed to understand their efficacy as a learning tool particularly in obstetrics and gynaecology. In conjunction with students, this study aims to develop a gynaecology-focused, evidence-based, medical education podcast and to assess its efficacy as a learning tool.

## Methods

### Study design

The design of this study was a sequential exploratory mixed-methods study with two phases. The first phase involved a descriptive qualitative study utilising focus group discussions with semi-structured questions to collect data regarding students’ preferences for an educational podcast. Informed by this student feedback, a 3-part gynaecology-focused podcast mini-series was designed and developed. The second phase of the study assessed the student’s perceptions of the podcast design, content and effectiveness as an educational tool using a questionnaire with a series of close-ended questions on 3–5 point Likert scales as well as questions with free-text responses for other feedback. This study design was underpinned by a pragmatic epistemological stance. Due to the busy student schedules and study time constraints, focus group sessions were utilised to enable a broad sampling of student experience and opinions that were later able to be assessed with the overall cohort feedback after listening to the podcast. The mixed-methods design was able to offer complementary insights into the practical use of podcasts as an educational tool. Ethical approval was granted by the University of Newcastle Human Ethics Advisory Panel (Approval number H-2023-0263).

### Participant recruitment and selection

Participants were recruited from the University of Newcastle and University of New England Joint Medical Program (JMP) in their 4th medical school year when they undertake a clinical placement in obstetrics and gynaecology. Students undertake their clinical placement at one of five clinical schools within Hunter New England Local Health district in New South Wales, Australia which include Newcastle, Maitland, Central Coast, Armidale and Tamworth. Education through this placement is delivered via a range of modalities including conventional lectures, case-based learning groups, clinical placement and online self-directed resources. In August of 2023, students were sent an invitation to participate in either the focus group and/or podcast evaluation arms of the study by email and the university online learning management system ‘Canvas’. There were no specific exclusion criteria, specifically current or previous podcast use was not a requirement to participate. Using convenience sampling, students from the total cohort of 201 who elected to participate were included in the study. Written informed consent was obtained from all participants prior to commencement of the study.

### Research team and reflexivity

All interviews were conducted by LR, a female trainee in Obstetrics and Gynaecology and Education Fellow at John Hunter Hospital. LR supervised and lead case-based learning tutorials with students on the Newcastle campus throughout their rotation. The purpose of the study was clearly outlined in the participant information statement and consent form. LR was supervised and mentored by GH, TG and BM-A, experienced qualitative researchers.

### Focus group qualitative data collection

A total of 3 focus groups (*n* = 14) were conducted and recorded over Zoom during a mutually agreed time. All focus groups were conducted by LR, using a semi-structured interview guide with open-ended questioning and took approximately 30 min each (Appendix 1). Participants were advised they could talk freely and safely about their opinions and experiences with podcast use. All sessions were audio-recorded over Zoom, transcribed verbatim and anonymised before analysis.

### Podcast development

Content from focus groups was used to guide the development of a gynaecology educational podcast 3-part mini-series entitled ‘Scrub Up’. Each podcast episode comprised a host (LR) and different specialist Gynaecologists, utilising a case-based approach to understand the work up and management of a common gynaecological condition which included urge incontinence, endometriosis and uterine fibroids. Based on student feedback from phase 1 of the study, the length of podcast was designed to be approximately 20–30 min long. The content of the podcast detailed the important aspects of history, examination, investigations and management strategies for each condition and were completed with a summary of key points at the end. Links to relevant guidelines or summaries were included in the podcast show notes.

### Podcast evaluation questionnaire

Participants in the podcast evaluation phase of the study were given access to the podcast (*n* = 82) and then asked to complete an evaluation questionnaire to complete after they had listened to the podcast. The evaluation questionnaire comprised a series of questions rated on a 3–5-point Likert scale to rate their experience of the content and quality of the podcast as a learning tool. The final question allowed for free-text responses and asked for any additional feedback. (Appendix 2.)

### Data analysis

Focus group data was analysed using an inductive thematic analysis method to derive meaning from the participant’s experience. Focus groups transcripts were read multiple times to understand the content and then subsequently analysed. LR first reviewed the data, identified concepts meaningful to the research question that were then labelled with a code and then formulated the first coding tree. Two additional researchers (TG and GH) reviewed the data and helped refine the coding tree through discussion and analysis. Data codes were organised into categories and from this a number of themes emerged. Themes were reviewed, reorganized and synthesized to create the final themes and findings with agreement from all three researchers. The results are reported according to the COREQ checklist [12].

Data from the participant enrolment and evaluation questionnaires were analysed using descriptive statistics.

## Results

A total of 82 (40.7%) students enrolled and consented to participate in the study. Fifteen participants elected to participate in the focus group session, although one of these participants later withdrew consent due to personal time constraints prior to participation and so 14 students (17.0%) participated in a focus group session. In the podcast evaluation arm of the study, 68 (33.8%) students accessed the podcast and 55 (27.3%) students completed the evaluation questionnaire.

Participants had an average age of 25.3 years. 34.1% of participants were male and 65.9% were female. Student participants were from across the different clinical school sites Newcastle 49 (59.8%), Maitland 12 (14.6%), Central coast 8 (9.8%), Armidale 10 (12.2%) and Tamworth 3 (3.7%).

At enrolment, 80.5% of the participants reported to already listen to podcasts, 61.0% listened to those focusing on medical education and of these, 64.0% listened to podcasts with gynaecological content.

### Focus group findings

Analysis of the focus group data revealed five main themes which captured the preferences of medical students in medical educational podcasts. These were: “personal and relatable” which “reflects real world clinical practice”, “use alongside conventional study”, “structured and focused content” and “targets what I need to know”. Within the first theme “personal and relatable”, a subtheme emerged of “learning from a different perspective”.

#### Personal and relatable

Podcasts were clearly differentiated by students from conventional study or lectures by the personal, conversational and engaging delivery of content which was strongly preferred by students. This allowed high level information or expertise to be more accessible and relatable to students.

*“Overall it felt a lot easier to engage with because it felt like they were just chatting”(FC2)*.

*“It feels like I’m learning with someone”(FB4)*

*“I think that’s why I like podcasts where there’s more than one person because then it feels like I’m having a conversation more than a one on one thing where I feel like I’m being told a lot of things.” (FB4)*.

*“They have conversational type things where they get experts in the field” (FA5)*.

Many podcasts were favoured for the story-telling style which took students along for a journey. The ‘human’ element of listening to a podcast, made students feel like their learning was more akin to a one-on-one mentorship or conversation which allowed it to be more relatable and memorable.

*“the way they put it into like a story probably sticks better*,* maybe four months down the road” (FA3)*.

*“Someone was like guiding the podcast and the other person was like doing the workup. And it was kind of like a long diagnostic journey” (FB1)*.

*“I like when they throw in random anecdotes*,* like ‘oh I had this patient’*,* it’s like relevant but a bit tangential. ‘I had this patient with a similar kind of thing’ I just find it a bit more engaging. I guess*,* from my perspective*,* if you take that sort of element out of it*,* there’s no real difference to reading a textbook. So I like it when it feels like you’re part of it and engaged.” (FB1)*.

When a presenter described the rationale for their clinical assessment and decision making this was a powerful learning experience for the listener, with many reporting this helped with information retention. When teachers described this rationale and nuance in their decision making, this was felt to be more valuable than the way information was often presented in a textbook or summary. Similarly, students valued when presenters used learning ‘hacks’ or pneumonics which they were able to utilise later in clinical practice or with study.

*“Its more about that insight and perspective of someone who’s in the field and working every day. That’s what would be really really valuable for me” (FB4)*.

*“Knowing the reasoning behind it means you don’t just rote learn things. You understand the reasoning*,* so then you know it*,* and use that moving forward” (FA5)*.

*“It was really engaging because they had really fun pneumonics throughout and little memory aids and how they remember things” (FC2)*.

*“really thinking about what you’re doing and why you’re doing it” (FB1)*.

Students often preferred more than one presenter (but not more than three) and indicated that the reason this number was best was to lean in to the more conversational and relatable tone. One person leading a podcast often lead to a ‘lecture-style’ presentation which was not as engaging to listeners.

*“I do like it when it’s at least two people like*,* I feel like one person podcast is very monologue” (FB4)*.

*“one person sometimes feels a bit like you being lectured” (FC1)*.

#### Reflects real-world clinical practice

Another theme that emerged from the focus group data was a preference for podcasts that used a clinical case example to guide the discussion in the podcast. Many students reported that this enhanced their understanding of the diagnostic process as well as facilitated their memory around a topic which was felt to be similar to seeing patients on their clinical placements. The use of real-world examples by presenters was felt by students to be highly valuable and more likely to assist them as clinicians in the future.

*“I love when it’s centred around a case or an example from practice. I feel like that really cements things for me.” (FC1)*.

*“one thing I like about a podcast*,* is the ones that I’ve listened to*,* they generally are using real cases” (FB1)*.

Many students reported that listening to podcasts where listeners were prompted to formulate an answer or management plan were particularly engaging for learning. This allowed students to feel like they were ‘in the shoes’ of the clinician and could actively consider the next management steps for themselves with real-time feedback.

*“at the same time you can kind of be thinking like*,* what would I do? And why? So that’s pretty good to kind of get you thinking.” (FA5)*.

*“I remember quite specific information from (the podcast) that I don’t remember from my study… and I think that’s because you got quizzed as you go” (FC2)*.

The structured nature of podcasts enabled listeners to feel that key concepts were captured and that the important evidence had been distilled and summarised in a digestible way. Particularly, students valued where podcasts alluded to the relevant clinical practice guidelines or summary articles to assist them with further learning on a topic.

***“****You’re not really sure what’s relevant or what’s actually used in practice. So having people tell you like ‘a guideline will say this*,* but this is what we actually do’ is really helpful” (FA5)*.

This desire for clinically relevant content was reflected in a near universal preference for Australian-based content when students selected a podcast. Students sought out content that was not only from reputable sources and speakers, but the choice of locally produced content enabled students to ensure that the summary of information would reflect the guidelines followed by clinicians and their University medical program.

*“I try not to listen to ones that aren’t Australian*,* because I just don’t want to get the wrong information*,* or not the wrong information*,* but the information that isn’t a part of our guidelines” (FA6)*.

*“Sometimes the pathways and the drugs or things they use will be different to the ones in Australia and they always use brand names…. So sometimes you’re not sure what they’re talking about. So I find that a bit confusing.” (FB2)*.

*“I try to pick things that I feel would be a little bit more like reputable. So either people I know like that are experts in that field or like the actual colleges” (FC1)*.

#### Use alongside conventional study

Most students described the way they utilised podcasts as an adjunct to their conventional study or preparation for clinical placement. Students utilised podcasts as a tool to save time and maximise their time to cover content that otherwise felt vast.

Students nearly uniformly reported that they engaged with podcasts for learning in times when conventional study wasn’t possible (while out walking, doing other household tasks or while commuting). When students felt they weren’t motivated to sit down and do conventional study, this was a way they could engage with content and feel they were still learning. The novelty of a different format of learning was additionally found to help with engagement around a topic.

*“when I don’t feel like sitting down at the desk*,* I can go for a walk and I feel like I’m doing something productive” (FA4)*.

*“sometimes…it’s been a long day*,* but I still have got to study something or do some pre-reading on a topic and I just am so over looking at a computer*,* my eyes are really tired*,* you know*,* have a headache. Maybe just listen to a podcast*,* but taking notes at the same time*,* is a really good way to actively learn. Still*,* without having to look at a jarring computer screen if you’re really over it. So I find that pretty helpful as well.” (FA5)*.

*“I never do the same method of study the whole way through the year. I’ve got to change it up*,* otherwise it just gets too monotonous…. a podcast is just adding to that. So its increasing the ways you can learn.”(FC2)*.

Many students reported that listening while commuting helped not only to feel like the time spent between home and placement was not wasted, but also helped to prepare them for the day ahead. This was particularly the case for students in placements in regional or rural areas where the travel time was longer.

“*I used them a lot to help just get through the content that I wasn’t covering well enough at home” (FC2)*.

*“I used to drive to (a rural placement) and back every week so I had 10 hours (on the drive). And I used to listen to a lot of them.” (FB1)*.

Podcasts importantly enabled students to maximise the use of their time which was limited. Many students reported feeling there was large amounts of content to cover without enough time, but listening to podcasts enabled them to engage with this information in an efficient and flexible way, often while doing other things.

*“But last year I was placed at (a rural clinical school) and with all the med and surg content we had to cover I was using them a lot to help with my learning*,* because I felt like I couldn’t get through stuff*,* and being almost a half hour drive I could almost do an episode each way.” (FC2)*.

#### Structured and focused content

Students clearly showed preference for a structured outline, a clear framework, and concise content delivery in their podcast choice. Students valued clear signposting about podcast content, and did not want to waste time finding the episode that would target what they wanted to know.

*“I’m more willing to click onto it if it’s specific with what you’re going to learn”(FA4)*.

*“I choose ones that are ‘to the point’” (FA2)*.

Students reported that podcasts often provided the learner with a clear framework with which to understand different medical concepts that they found helpful to take into real world clinical practice. Such a structured approach helped to consolidate their learning. Many liked if the podcast structure was segmented in design and enabled a summary of key points.

*‘Introducing the topic, and then the investigation and management, it’s a nice little package all tied up with everything you need to know about a topic’ (FC1)*.

*“I find I pick up more when there might be a stop. So if it’s a 30 min podcast, every 10 min or so to just stop and say, ‘okay, so we just covered that’ or ‘just going over what we’ve quickly covered’, just like highlighting the salient points.” (FC2)*.

Students preferred episodes to be brief and to the point which reflected their need for efficiency in how they engaged with learning. This often reflected the time they wanted to be listening, with the ideal length of time for a podcast often reflecting the time it took students to commute back and forth to placement.

*“Going to placement, it’s only about a 15 min drive for me, so I want something sort of short, because I don’t want to be picking it back up like halfway through on the way home and forgetting for what happened at the start and being a bit lost.” (FB3)*.

*“I think 30 min pretty good. II feel like most of my commutes day to day are that kind of like 20 to 30 min.“ (FC1)*.

Students reported that the use of direct and informative language provided them with the essence of a topic that then enabled them a better understanding of the topic. Many students reported that a strong deterrent for them was when a presenter lacked ability to summarise the important and relevant information.

*“not too much waffle” (FA2)*

*“if they end up getting a bit sidetracked eventually*,* you kind of are just like*,* oh*,* well*,* this is missing the point a bit.” (FC2)*.

#### Targets what I need to know

The final theme was how students chose podcasts that were specifically targeting their learning needs. Many preferred podcasts that reached the right level of detail, and level of language that could be understood by a student. Podcasts with language or content that was too complex or obscure were avoided.

“*more tailored to a medical student knowledge” (FB2)*

*“giving you all the main points you need to know as a student” (FA5)*.

*“I don’t want extra content that’s too hard for me” (FB2)*.

Interestingly, there was some differentiation between the learning needs they had as students compared with the learning needs required to enter clinical practice. Many students reported seeking out content that matched the learning curriculum and commonly examined topics to help them with revising for exams. They described these topics as high “yield” and more likely to assist them with better results.

*“big topics that we learned within our CBL (case based learning)*,* those were the ones that I was searching for” (FA5)*.

*“you can have an … undifferentiated presentation at triage and that sort of going through the history. And jog my memory for how I’d do it for exams. So that’s why I prefer it” (FB1)*.

Although the learning points prescribed within university medical education are intended to reflect clinical practice, students reported that they often listened to podcasts for the insights they might get into working as a clinician independent from content that helps with study. Students expressed that certain podcasts were able to reveal nuance and a level of practicality that conventional education couldn’t reach that would in the end make them a better doctor.

*“they generally are using real cases. So there’ll be, you know, red herrings and there’ll be atypical features on ultrasound, and that kind of thing, and it kind of I think, it’s training you to be a better doctor” (FB1)*.

### Utilisation of focus group data for creation of ‘Scrub up’

Participant feedback from focus groups were used to inform the creation of a 3-part mini-series. Utilising the main themes derived from the focus groups, a 3-part mini series podcast focused on gynaecological topics were created. At the request of students for topics that were common and clinically relevant, the podcasts focused on urinary incontinence, endometriosis and uterine fibroids. The design of the podcast was utilising a clinical case in order to discuss the investigation and management of the condition. Using an interview-style with 2 presenters (LR and a specialist gynaecologist), the tone of the podcast was intentionally casual and conversational, employing examples, pneumonics, repetition, summaries and other tools that students had reported to enhance their engagement and knowledge acquisition while listening. Based on focus group feedback, a clear structure was created, with breaks for each section to ensure the podcast was easy to follow.

### Evaluation questionnaire results

**Fig. 1:**
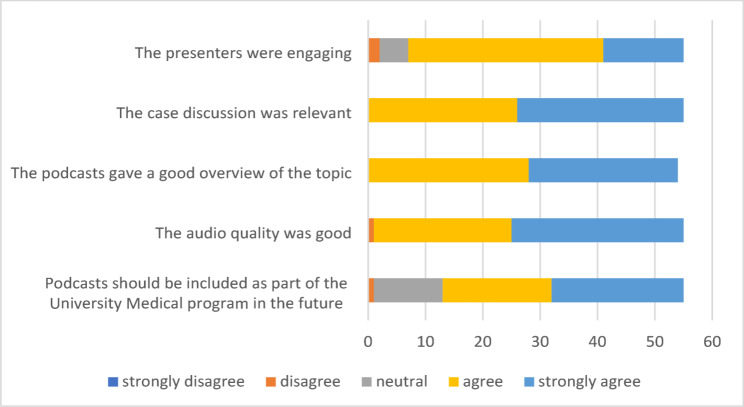
Podcast evaluation questionnaire responses

Once participants listened to the ‘Scrub Up’ podcast, they completed an evaluation questionnaire and results of this are included in Fig. 1. Overall, 92.7% of participants felt the podcasts were effective or very effective at improving their knowledge in the gynaecology topic areas. Participants felt the length of the podcast to be just right 89.1% of the time and the level of content to be just right 85.5% of the time. Respondents felt that the podcast presenters were engaging (strongly agree 25.5%, agree 61.8%), gave relevant case discussions (strongly agree 52.7%, agree 47.3%), a good overview of the topic (strongly agree 47.3%, agree 51.9%) and that the audio quality was good (strongly agree 54.5%, agree 43.6%). The vast majority of students agreed (34.5%) or strongly agreed (41.8%) that podcasts should be included as part of the university medical program in the future. Feedback was similar for participants who reported to have not previously engaged with podcasts.

Twenty-nine participants provided additional comments. Of these, 20 of the comments provided positive support of the utility of the podcast, namely that the content was relevant, the structure was clear, the presenters engaging and that the content was very useful for revision of topics for their exam revision. Some students reported wanting more, or the content to be expanded to include obstetric cases.

*“The overall format was engaging and formatted in a clear way. Very useful as a study tool for structured study but also for enjoyment.”*

*“A well produced podcast, relevant and engaging guests, important clinical knowledge and good use of case vignettes!”*

*“These were great podcasts and very informative! I loved the jingle as well! It was really valuable to have a resource on topics that are important for O&G but ones that we don’t have time to discuss in class. Thanks for making them!”*

*“Good way to learn. Would consider these more useful than pre-recorded lectures that we have access to.”*

Sixteen of the comments gave suggestions for improvements which included comments on the length or level of detail. Some listeners requested more details of the case to assist with their engagement in the topic and others requested more clinician anecdotes, and learning pneumonics to help with memorising content.

*“Just to make it more centered around the people.”*

*“consider making it more of a diagnostic challenge for the students to get them practicing their work ups.”*

“*Maybe include exam type questions or highlight ‘clinical pearls’ after the end of sections.*”

*“It was obvious some guest doctors were reading a script. understandable as they’re not professional podcaster but would be more engaging if they talked without script. Otherwise, thanks for the OSCE prep!”*

There were four comments expressing that podcasts should be included as a resource for students but didn’t want this content to be made mandatory given they felt that not everyone would like and utilise podcasts in the same way.

*“Podcasts make it difficult for students with hearing deficiencies, attention deficits and other neurodivergent traits to engage with the topic. They should only be offered as an adjunct to standard teaching.”*

*“Podcast should be included as tools for students, but they should not be mandatory classes to listen to as not everyone benefits from this type of learning.”*

## Discussion

Our study intended to explore the attitudes of medical students towards podcasts as a learning tool and with this knowledge develop a tailor-made podcast series to meet these learning needs. In doing so, we hoped to better understand how students can harness the benefits of this novel educational tool.

In our study, based on focus group data, podcasts were reported by students to be a flexible, accessible, targeted learning tool for students. Students described engaging with podcasts as an adjunct to conventional educational tools as well as helping them use their time more efficiently when doing other things. This is consistent with previous research showing that learners often utilise podcasts while multi-tasking (driving, exercising and doing chores) to supplement rather than replace other learning modalities [13]. Knowledge retention from listening to podcasts while commuting and exercising has been shown to be equivalent to listening while in a quiet room. and is therefore a way of maximising the use of these otherwise unproductive times [14],^15^.Further, the order that the information was delivered within podcasts did not seem to affect knowledge retention [15]. For clinicians, managing scarce time and multiple competing priorities means that asynchronous access to podcasts can enhance the use of time to engage in learning and potentially replace didactic video or face to face lectures.

Students in our study valued the relaxed, relatable delivery of podcasts which increased their engagement in a topic. Numerous studies have previously reported that learners across medical specialties value the conversational tone and dialogue of podcasts which makes listening entertaining and enjoyable [3]. Podcasts provide the benefits of reducing stress that might otherwise occur in more traditional learning contexts, such as talking to an expert in a lecture theatre or practicing for examinations in front of peers and teachers [6]. The social phenomenon of podcasting has been explained by Riddel et al. as enabling listeners to feel like they are part of a community and more connected to their peers, supervisors and larger professional community through an understanding of the common language and challenges of the specialty [16].

Students in our study reported that the use of clinical cases and real-world experience was a quality of podcasts that assisted with knowledge acquisition. This is consistent with previous literature including one study assessing a neurology podcast revealed that even when compared with written case examples, podcasts were felt to be a more engaging and enjoyable learning experience with equivalent knowledge acquisition [17]. The use of real world context may also allow podcasts to benefit learners beyond knowledge dissemination through role modelling cultural competence and the use of critical thinking which are medical concepts that are difficult to learn by reading a textbook [6].

The main strength of the study was the mixed methods design that enabled utilisation of student insights from qualitative data to inform the podcast creation. Focus groups provided the ideal setting for generation of ideas and free expression of their views on podcast design and content preferences. Opinions from students about the length, style, tone and content focus of the podcasts as described in the focus group reviews were utilised in order to create tailor-made content for the students. This unique dialogue between educational developer and learner provided a successful modality to help tailor the way information is communicated in order to optimise learning outcomes.

Our study demonstrates that podcasts are a feasible tool for medical educators to attempt. The authors, new to the creation of podcasts, were rapidly able to acquire the skills to create the final content (approximately 2–3 h content preparation, 1 h recording and 1–2 h editing) and this efficiency improved with each episode. Other studies report a range of 2–15 h preparation time with low associated costs at commencement of podcast production [9].

Our study was subject to some limitations. We were able to enrol 40% of all students in the cohort in our study. It is therefore possible that feedback was from students who were more inclined to already have listened to and engaged with podcasts and our findings may not be generalisable to all students. Further, it was not possible due to limitations in the podcast software used to know if students completed listening to the podcast or the time to evaluation which may impact on the responses provided. However, studies have consistently shown that a high proportion of clinicians and students engage with podcasts and preference them as a modality for learning and our findings are consistent with this [3].

A recent systematic review examining the utilisation and efficacy of podcasts for medical education demonstrated that despite increasing uptake of podcasts, high quality research evaluating performance in examinations or longer term knowledge retention remain limited [9]. Future research could focus on assessing the impact of podcast learning on exam performance, knowledge retention and clinical practice. Further, understanding how the learning needs of clinicians differs to that of medical students could enable creation of podcasts to better target the learning needs of obstetric and gynaecological trainees and consultants.

## Conclusions

Medical educational podcasts are used widely amongst medical students and doctors to supplement learning and keep abreast with evidence-based medicine. They serve as a flexible, highly effective adjuncts to conventional medical educational tools. The specialty of obstetrics and gynaecology particularly lends itself to utilisation of flexible learning tools such as podcasts due to the demanding and fluctuating workload of clinicians. This research provides evidence to help understand how best to harness podcasts to target the learning needs of students and doctors and to design tailored content to support our medical workforce of the future.

## Data Availability

The datasets generated and/or analysed during the current study are not publicly available but are available from the corresponding author on reasonable request.

## Appendix 1

Focus group interview schedule

1. Tell me about your experiences currently listening to podcasts?
  - Prompts: What content/topics do you listen to? when do you listen? how do you choose what to listen to?

2. Why do you listen to podcasts? What do you think you gain from listening to podcasts that are different to conventional education delivery?
  - Prompts: How do they help you to understand a topic? What are unique to podcasts compared with conventional education? Flexibility of listening time, changing listening speed, listening multiple times…

3. What features do you or would you like in a podcast?
  - Prompts: presenters, audio quality, length, content: cases, summaries, robust discussion, simple

4. What features do you not or would you not like in a podcast?
5. Do you easily remember content that you have listened to?
  - Prompts: what type of learner are you?

6. Would you listen to a podcast focused on Gynaecological topics?
  - Prompts: If no, why not? If yes, how do you think this would benefit your learning?

7. What aspects of gynaecology do you think would be best covered in a podcast? Why?
8. Do you have anything further you would like to tell me, ask me or talk about?

## Appendix 2

Evaluation questionnaire

1. Rate the overall effectiveness of this podcast for improving your knowledge on this gynaecological topic.
  a 1 = very ineffective; 2 = ineffective; 3 = neutral; 4 = effective; 5 = very effective

2. The challenge of understanding the content in these podcasts were:
  a 1= too little, 2= just right, 3 = too much

3. The length of the podcast was:
  a 1= too short, 2 = just right, 3 = too long

4. Please indicate your level of agreement with the following statements:
  a The presenters were engaging
    - 1=strongly disagree, 2= disagree, 3=neutral, 4=agree, 5=strongly agree
  b The case discussion was relevant
    - 1=strongly disagree, 2= disagree, 3=neutral, 4=agree, 5=strongly agree
  c It gave a good overview of the topic
    - 1=strongly disagree, 2=disagree, 3=neutral, 4=agree, 5=strongly agree
  d The audio quality was good
    - 1=strongly disagree, 2= disagree, 3=neutral, 4=agree, 5=strongly agree
  e Podcasts should be included as part of the University medical program in the future
    - 1=strongly disagree, 2= disagree, 3=neutral, 4=agree, 5=strongly agree

5. Any other feedback you would like to provide to help us improve this podcast for the future? (Free text)
